# Hematological Evaluation of Three Common Teleosts in Relation to The Environmental Changes from Trang Province, Thailand

**DOI:** 10.21315/tlsr2023.34.3.6

**Published:** 2023-09-30

**Authors:** Archig Jeamah, Sinlapachai Senarat, Suparat Kong-oh, Chanyut Sudtongkong, Porntep Wirachwong, Natthawut Charoenphon, Nontawat Kawjaeng, Pahol Kosiyachinda, Anan Kenthao, Piyakorn Boonyoung

**Affiliations:** 1 Department of Marine Science and Environment, Faculty of Science and Fisheries Technology, Rajamangala University of Technology Srivijaya, Trang 92150, Thailand; 2 Division of Biological Science, Faculty of Science, Prince of Songkhla University, Songkhla 90110, Thailand; 3 Department of Anatomy, Faculty of Medical Science, Naresuan University, Phitsanulok 65000 Thailand; 4 Department of Biology, Faculty of Science, Mahidol University, Ratchathewi, Bangkok 10400 Thailand; 5 Department of Biology, Faculty of Science, Naresuan University, Phitsanulok 65000 Thailand; 6 Division of Health and Applied Sciences, Faculty of Science, Prince of Songkla University, Songkhla 90112 Thailand

**Keywords:** Sentinel Species, Marine Environmental Monitoring, Estuarine Fishes, Fish Hematology, Marine Conservation

## Abstract

Hematological evaluation of fish is essential to the assessment of their physiological status. This study describes the morphometric analysis and comparison of blood cell characteristics in *Zanarchopterus* sp., *Gerres filamentosus* Cuvier, 1829 and *Leiognathus decorus* (De Vis, 1884). The species were collected at two locations off the coast of Trang Province, Thailand. A comparative hematological evaluation was made to assess the effects of environmental conditions on the blood of the fish. Ten individuals of each species were collected from a seagrass bed at Libong Island, where human activities are increasing, and from a secluded sandy beach. Their blood samples were analysed using the blood smear technique. Erythrocytes of all the studied fishes were either elliptical or oval. The morphometric data from both locations showed that erythrocytes were of similar size, except for those of *Zanarchopterus* sp. Fish from both stations showed several types of leukocytes, including neutrophils and lymphocytes.The highest proportion of leukocytes was made up of lymphocytes, followed by neutrophils. However, monocytes were only observed in fish from Libong Island and the erythrocytic nuclei of fish collected from Libong Island were both reniform and lobate. Our results show the potential of hematological evaluation as an early warning signal of environmental impacts on aquatic animals. The determination of baseline parameters could provide a tool for the monitoring of environmental quality.

**Highlights**

- Evaluation and characteristics of blood cells from representative coastal areas as well as the seagrass bed of Thailand.
- Erythrocytic nuclear abnormalities and percent proportions of leucocytes of fishes were found especially the seagrass bed.
- The potential environmental health problems at Libong Island have been proposed.

## INTRODUCTION

Marine ecosystems are complex with a high level of biodiversity. The marine environment varies greatly from one location to another and the local ecological structure can be influenced by, for example, sandy beaches, mangrove forests, and seagrass reefs (Cheevaporn & Menasveta 2003; Pradit 2020; Wattayakorn 2012). In marine ecosystems, aquatic animals build complex food webs as they exploit the available resources. In the Asia-Pacific region, moreover, they contribute significantly to national economies (Cheevaporn & Menasveta 2003; Wattayakorn 2012). The capture fisheries and aquaculture sectors generate income for coastal communities and are critical to national food security. Current commercial fishing conditions, and the development of large-scale coastal aquaculture and tourism, have resulted in environmental problems and pollution in coastal regions (Cheevaporn & Menasveta 2003; Wattayakorn 2012). In addition, various remote ecological changes, including deforestation and industrialisation, have destroyed and altered ecosystems, leading to the loss of biodiversity in coastal areas of Thailand.

The monitoring of environmental problems has long been assisted by the use of sentinel species. Fish species are studied as sentinel species or components of animal sentinel systems because they are plentiful, easy to handle, and tolerate a wide range of environmental conditions (Beeby 2001). The most useful species respond to specific pollutants (Carpenter 1925). The effects of a contaminant when it enters the body of an animal are demonstrated by biological markers. Commonly used biological markers are organ weights but hematological markers are numerous (Auró de Ocampo & Ocampo 1999; Kendall *et al*. 2001). Numbers of red blood cells (RBCs), white blood cells (WBCs), and thrombocytes are typically used to assess the health status of fish (Clauss *et al*. 2008; Nussey *et al*. 1995). Changes in blood cell size and shape, and nuclear alterations can be physiological indicators of sensitivity to environmental stresses (Ergene *et al*. 2007; Megarani *et al*. 2020; Parrino *et al*. 2018), and the size and volume of erythrocytes, can imply the physiological adaptation of fish to their environment (Campbell & Murru 1990; Dal’Bó *et al*. 2015; Koldkjaer & Berenbrink 2007; Lay & Baldwin 1999; Witeska 2013).

Recently, the coastal areas of Trang Province in Thailand have been recognised for their complex ecological compositions. At the same time, the promotion of tourism in the province by the government has led to increased anthropogenic activities, especially around Libong Island. Inevitably, the issue of marine pollution from waste has had a significant impact on the environment in this area (Pradit 2020). Many types of hazardous waste, including batteries, were recently reported to have been disposed of in the sea (Pradit 2020). Besides tourism, the rubber industry, agriculture and household activities cause further environmental problems. The resulting pollutants can accumulate in aquatic animals and people who consume them (Pradit 2020).

The goal of the present study is to characterise the features and determine the morphometry of blood cells in three pelagic fish species, *Gerres filamentosus* Cuvier, 1829, *Leiognathus decorus* (De Vis, 1884) and *Zanarchopterus* sp. collected at a seagrass bed near Libong Island and off a secluded sandy beach on the mainland coast of Trang Province, Thailand. All fishes were used as sentinel species, which have been followed by the previous characterisers above. This study will shed light on the physiological response of these fish to the environmental situation in their habitats. The results have implications for the environmental health status of the area and the relationship between aquatic animal health and human health in the area.

## MATERIALS AND METHODS

### Study Areas and Fish Collections

Three pelagic fish species, *Gerres filamentosus* Cuvier, 1829, *Leiognathus decorus* (De Vis, 1884) and *Zanarchopterus* sp. that are common and a wide range in this coastal area of Trang province, Thailand were selected as sentinel species in this study. Ten individuals of each species were collected from two locations in Trang Province, Thailand: a seagrass reef in the Libong Island area (geographical coordinates) and open waters off a sandy mainland beach (geographical coordinates) known as Rajamangkala. The specimens were obtained by trawling and netting in June and July 2021. During the collection of the specimens, multiparameter measurements (U-50 – Horiba, HORIBA Advanced Techno Co., Ltd., Japan) were taken to obtain environmental parameters of dissolved oxygen (DO), and water temperature, salinity and pH. Ethical approval for the work was provided by the Animal Care and Use Committee of Rajamagala University of Technology Srivijaya (ID#IAC 13-04-64).

### Morphological Measurements and Analysis of Blood Cells

Collected specimens were immediately subjected to a rapid cooling shock at 2°C–4°C by submersion in water mixed with ice at a ratio of 1:1 (Wilson *et al*. 2009). All fish were measured for their total length (TL) and total weight (TW). Blood samples of 0.3 mL–0.5 mL were taken from 10 fish per species/region, drawn from the heart area with a 1 mL plastic syringe, needle No. 21G. Following the standard method of Singkhanan *et al*. (2019), approximately 10 μL of each sample were smeared on a glass slide and left to dry at room temperature, forming a thin film. The prepared slides were treated with methyl alcohol for 1 min, stained with Wright’s Giemsa for 15–25 min, and the dye was then washed off with a pH 7.4 buffer solution for 30 min. The morphology of blood cells on the slides was then studied (Cossins & Gibson 1997). The blood cell typing and morphological analysis including erythrocyte length (EW), erythrocyte width (EL), nucleus length (NL) and nucleus width (NW), blood cell percentages, and blood cell abnormalities were evaluated according to the standards of Andreyeva and Mukhanov (2012), which were performed using Olympus BX53 light microscope (Olympus Corporation, Tokyo, Japan). Total abnormal erythrocytes (from 100 cells/fish per slide) and leucocytes (from 10 cells/fish per slide) were counted, and then calculated as percentages.

### Statistical Analysis

The morphometric data of blood cells were presented as means ± standard deviations (SD). The normal distribution of our results was checked with the Kolmogorov-Smirnov Test (K-S Test). Hoteling’s T2 test and independent *t*-test one-way Analysis of Variance (ANOVA) were performed and significant differences were determined by Duncan’s posthoc test at the significance level of *P* < 0.05. Sequential univariate analysis of the data was managed by SPSS version 22.

## RESULTS

### Environmental Factors

The environmental parameters measured at each collection station were compared. Although salinity and DO values were significantly different between the two locations (*P* < 0.05, Table 1), the values of all environmental parameter were within the range of standard values for marine environmental resources (Pollution Control Department 2017).

### Morphological Characterisation and Morphometric Analysis of Erythrocytes

**Figure 1:**
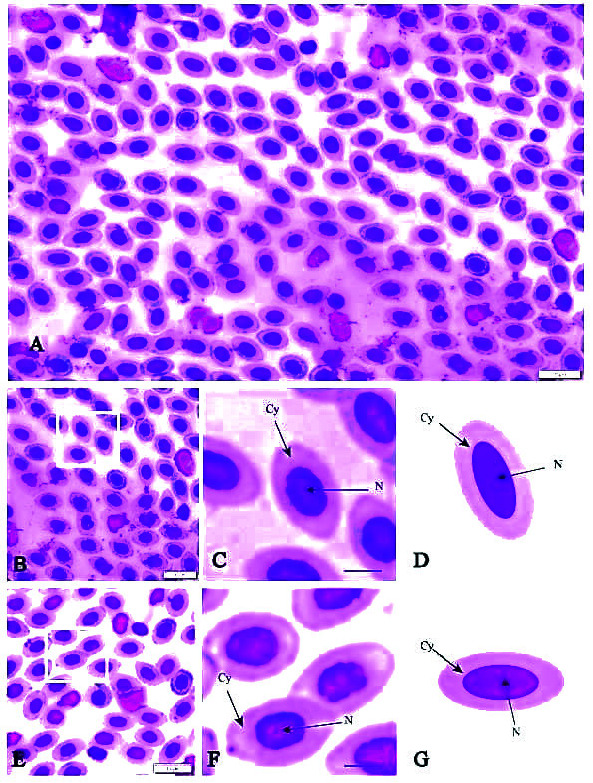
Blood samples were taken from fish collected at Rajamangala Beach and Libong Island, Trang, Thailand. Erythrocytes from the samples were morphologically compared. (A) Overall peripheral blood cells and erythrocytes. Micrographs (B) and, in higher magnification, (C) show the representative morphology of erythrocytes from *Gerres filamentosus* collected at Rajamangala Beach, with a schematic diagram (D). Images E–G show the same features of *Zanarchopterus* sp from Libong Island. Abbreviations: Cy = cytoplasm, N = nucleus.

The erythrocyte was the most abundant cell type of all specimens The most mature erythrocytes were either oval or elliptical in shape (Figs. 1A–1D). Erythrocyte nuclei were oval to spherical and surrounded by a pink cytoplasm (Figs. 1B–1G). Reticulocytes were not detected throughout the analysis.

Studies of erythrocyte morphometry showed that *Zanarchopterus* sp. from Libong Island exhibited the longest EL (8.49 ± 0.80 μm) and *L. decorus* from Libong Island exhibited the shortest EL (6.15 ± 0.68 μm). The largest EW was also observed in *Zanarchopterus* sp. from Libong Island (6.45 ± 0.70 μm), while the shortest EW was observed in *Zanarchopterus* sp. collected from Rajamangala Beach (4.53 ± 0.70 μm). Erythrocyte NL ranged from 2.87 μm to 3.47 μm. The longest and shortest values were observed in *L. decorus* (3.46 ± 0.61 μm) and *Zanarchopterus* sp. (2.87 ± 0.55 μm) both collected off Rajamangala Beach. The highest value for NW was observed in *Zanarchopterus* sp. from Libong Island (3.16 ± 0.50 μm), while the lowest value was found in *Zanarchopterus* sp. from Rajamangala Beach (2.21 ± 0.50 μm) (Table 2).

Multivariate analysis indicated that there was a significant statistical difference between the average size of erythrocytes of *Zanarchopterus* sp, collected from the Rajamangala Beach site and *Zanarchopterus* sp, collected from the Libong Island site. However, there was no such statistical difference in the overall size of erythrocytes of *G. filamentosus* and *L. decorus*. The result was verified by a series of independent *t* tests. The tests also indicated a significant difference in the average values of the four erythrocyte parameters between *Zanarchopterus* sp from the two habitats (Table 2).

### Morphological Characterisation and Percent Proportion of Abnormal Erythrocytes

**Figure 2:**
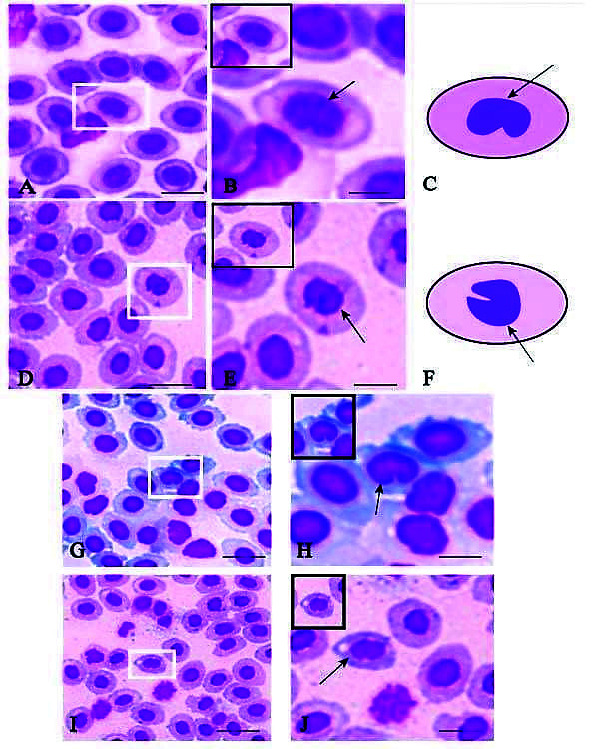
Erythrocyte abnormalities in blood of fish collected at Rajamangala Beach and Libong Island. (A–C) show reniform nuclei of *Zanarchopterus* sp from Rajamangala Beach; (D–F) show notched nuclei of *Zanarchopterus* sp. from Rajamangala Beach. (G–H) show reniform nuclei of *Gerres filamentosus* from Libong Island; (I–J) show notched nuclei of *Gerres filamentosus* from Libong Island.

Morphological abnormalities of the erythrocyte nucleus were observed in all three species from Rajamangala Beach and Libong Island. Two common erythrocyte abnormalities were kidney-shaped, or reniform, nuclei and lobed nuclei (Figs. 2A–2J).

The highest proportion of kidney-shaped nuclei was observed in *G. filamentosus* from Libong Island (3.00%) and the lowest proportion was observed in *G. filamentosus* from Rajamangala Beach (1.4%).

The largest percentage of nuclei with notches was seen in *G. Filamentosus* (3.52%) from Rajamangala Beach (Table 3). Our findings suggest that the fish from Libong Island have a higher percentage of abnormal erythrocyte nuclei than the fish from Rajamangala Beach.

### Comparative Characterisation and Morphometric Analysis of Leucocytes

**Figure 3:**
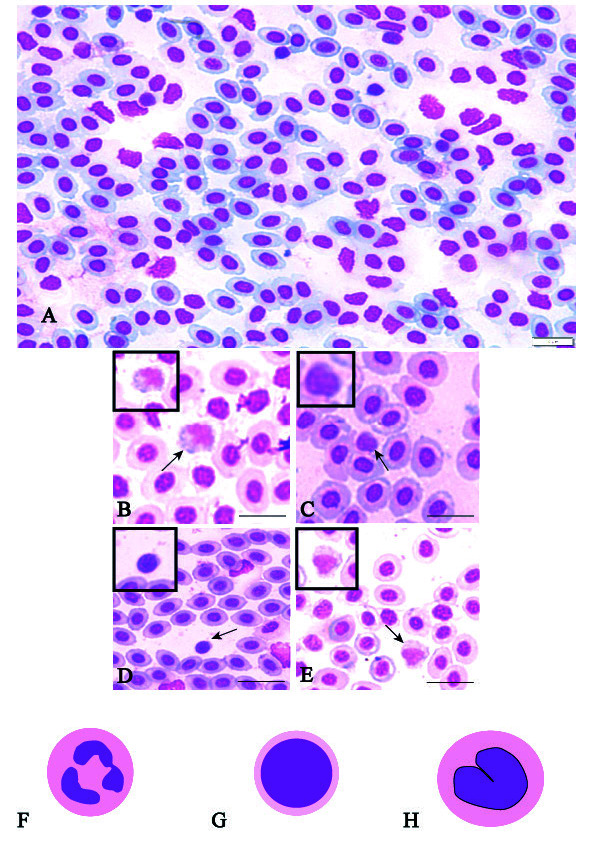
Leucocytes of fish collected at Rajamangala Beach and Libong Island, Trang, Thailand. Image (A) shows peripheral blood cells with the presence of leucocytes among erythrocytes in *Leiognathus decorus* from Rajamangala Beach, while (B) and (C) show neutrophils and lymphocytes. Several representative leucocytes lymphocytes (D) and monocytes (E) can be seen in blood from *Gerres filamentosus* from Libong Island. Illustrations (F), (G) and (H) depict a neutrophil, lymphocyte and monocyte, respectively.

Our comparative study revealed three major types of leucocytes present in the blood of the studied species (Fig. 3A): neutrophils, lymphocytes and monocytes (Table 4). Generally, the neutrophils were spherical with multi-lobed nuclei (Figs. 3B, 3F). The cytoplasm was purplish, having light purple-stained granules (Figs. 3B). Lymphocytes had a large central nucleus, surrounded by scanty, lightly stained cytoplasm (Figs. 3C–3D, 3G). The ovoid monocytes were distinguished by a large eccentric nucleus. They usually appeared as a slightly basophilic nucleoplasm (Figs. 3E, 3H).

The highest proportion of neutrophils (3.33%) was found in *G. filamentosus* from Libong Island, the highest proportion of lymphocytes (6%) was found in *L. decorus* from Rajamangala Beach, and the highest proportion of monocytes (3%) was observed in *Zanarchopterus* sp. From Libong Island (Table 4).

## DISCUSSION

The physiological dependence of fish on their environment brings them into an extremely close relationship with their surroundings (Acharya & Mohanty 2014; Giannetto *et al*. 2014; Parrino *et al*. 2018). It is well established that environmental factors affect their blood cell numbers, distribution and morphology (Maciak & Kostelecka-Myrcha 2011; Megarani *et al*. 2020; Parrino *et al*. 2018). Therefore, hematological parameters are widely used as an early indicator of changes in the health status of fish and have proven to be an effective method for monitoring the effects of habitat changes on fish biology (Fazio *et al*. 2013; Gabriel *et al*. 2004; Parrino *et al*. 2018).

External environmental factors and an organism’s energy requirements have a significant effect on hemoglobin concentration, the size and number of erythrocytes, and subsequently, the total surface area of blood cells in circulation (Maciak & Kostelecka-Myrcha 2011). Changes in the size of erythrocytes are inversely proportional to the metabolic activity of an organism (Smith 1925). Therefore, any adaptive decrease in metabolic rate would be followed by a tendency for the cell size to increase (Dal’Bó *et al*. 2015; Holland 1970; Szarski 1970; Val 2000; Witeska 2013). It has also been established that erythrocyte contents determine the capacity of an organism to carry dissolved oxygen (Holland 1970; Val 2000; Witeska 2013). From our analysis of erythrocyte morphometry among the species studied, only *Zanarchopterus* sp. showed significant differences between the two study sites. Previous studies of differences in physiological activities of fish indicated that the rate of oxygen consumption was inversely related to the volume of erythrocytes (Maciak & Kostelecka-Myrcha 2011; Longeville & Stingaciu 2017; Parrino *et al*. 2018). We propose that the benefits of increased erythrocyte size, which was found exclusively in *Zanarchopterus* sp. from Libong Island, must be weighed against the costs of limiting oxygen intake and transport. The amount of oxygen carried by erythrocytes in a unit of time is proportional to the rate of blood flow (Nikinmaa 2002), but the efficiency of gas exchange is proportional to the rate of water flow through the gills (Evans *et al*. 2005). As a result, the greater surface area to size of erythrocytes observed in *Zanarchopterus* sp from Libong Island may allow faster oxygenation and deoxygenation of hemoglobin to the extent that erythrocyte volume declines. This conclusion would also be consistent with the findings of previous investigations (Holland & Forster 1966; Jones 1979; Richardson & Swietach 2016).

Nuclear abnormalities in fish erythrocytes have been well observed and studied by Carrasco *et al*. (1990), who suggested that affected fish possibly suffered reduced respirational function. Since then, abnormalities of erythrocyte nuclei in fish have been widely used as a biomarker of various contaminant exposures for different fish species. The method is simple, sensitive, rapid and inexpensive (Carrasco *et al*. 1990). Here, we investigated erythrocytic nuclear abnormalities between two stations and found reniform and lobed nuclei. These abnormalities might be impacts of environmental problems, such as heavy metal toxicity (such as copper and cadmium) (Guner *et al*. 2011; Kousar & Javed 2015) and genotoxic damage (Kousar & Javed 2015; Summak *et al*. 2010). The possible reasons above will be investigated in further studies.

The comparative data showed that the highest proportion of leukocytes of fish from both stations was made up of lymphocytes, with the most abundant number found in specimens collected at Libong Island. In the work of Murtha *et al*. (2003), the percent proportion of lymphocytes in leukocytes of zebrafish reached 71%–92%. However, it has been proposed that variations in components of leukocytes in vertebrate species might be associated with age, season, and habitat conditions (Dessauer 1970; Duguy 2010; Hutchison & Szarski 1965). We observed monocytes only in leukocytes of fish from Libong Island. Although the function of monocytes has not been well described for fish, their general roles involve infection, inflammation, and tissue injury (Okabe & Medzhitov 2016; Clauss *et al*. 2008). It has been reported that monocytes also have important functions in cytokine production (Secombes 1991). Afiyanti *et al*. (2018) proposed that increased numbers of monocytes were associated with bacterial infection. Nussey *et al*. (1995) generally found that leucocyte counts were higher in contaminated fish. More recently, da Silva Correa *et al*. (2017) reported that increased numbers of monocytes were associated with unfavorable environmental conditions and polluted locations. Our findings suggest the appearance of some leukocytes of the fish from Libong Island that might have been exposed to contaminations or other environmental problems. The existence of environmental problems in the studied area is also implied by the known loss of local seagrass meadows. Observations of the seagrass beds around Libong Island, in particular, have indicated the threat posed by anthropogenic activities. The dredging of the Marine Department’s channel produced a large amount of sediment, which was then leached in fresh water and released back into the sea. The loss of seagrass meadows in some areas around Libong Island has since been linked to sediment deposition (https://www.dmcr.go.th/detailAll/49292/nws/22). As a result of this environmental change, the immune response of fish in this area might potentially have been stimulated.

## CONCLUSION

Characteristics of fish blood cells from representative coastal areas of Trang Province, Thailand were reported here for the first time. The blood of fish from a seagrass bed at Libong Island, where human activities are increasing, and the blood of fish from a secluded sandy beach of Rajamangala Beach were analysed and compared. Erythrocytic nuclear abnormalities and percent proportions of leucocytes indicated early manifestations of potential environmental health problems at Libong Island. Our findings may lead to the development of indicators, such as baseline levels of abnormalities, that can identify the effects of environmental changes on organisms in coastal areas. Hematological evaluation of fish could enable the early detection of pollutants and provide information about water quality and assist environmental protection or restoration efforts in impacted areas.

## Figures and Tables

**Table 1:** Observation of environmental factors from Rajamangala Beach and Libong Island.

Water physicochemical parameter	Rajamangala Beach	Libong Island	Reference (Lohaluksanadech *et al*. 2008)
Temperature	31.69 ± 0.13*	30.98 ± 0.08*	29.25–30.61
pH	7.67 ± 0.02	7.62 ± 0.05	7.74–8.18
Salinity	29.97 ± 0.23*	31.10 ± 0.17*	19.67–30.46
DO	8.01 ± 0.10*	5.94 ± 0.13*	5.63–6.98

**Table 2:** Six morphometric measurements of erythrocytes from 1,000 total cells of *Zanarchopterus* sp., *Gerres filamentosus* and *Leiognathus decorus*.

Stations	Positions (μm)	*Zanarchopterus* sp.	*Gerres filamentosus*	*Leiognathus decorus*
Rajamangala Beach	NL (N1)	2.87 ± 0.55*	3.45 ± 0.50	3.46 ± 0.61
	NW (N2)	2.21 ± 0.50*	2.40 ± 0.45	2.64 ± 0.57
	EL (C1)	7.62 ± 0.99*	7.61 ± 0.60	6.27 ± 1.32
	EW (C2)	4.53 ± 0.66*	4.67 ± 0.74	4.71 ± 0.86
Libong Island	NL (N1)	3.41 ± 0.83*	3.36 ± 0.43	3.13 ± 0.41
	NW (N2)	3.16 ± 0.50*	2.27 ± 0.40	2.86 ± 0.53
	EL (C1)	8.49 ± 0.80*	6.15 ± 0.68	6.85 ± 0.80
	EW C2	6.45 ± 0.70*	4.61 ± 0.63	5.35 ± 0.61

*Note*: EL: Erythrocyte Length, EW: Erythrocyte Width, NL: Nucleus Length, NW: Nucleus Width. The letter a, b, c and d indicate significant difference (*P* < 0.05) when the data are compared with other groups.

**Table 3:** Abnormality percentages of erythrocyte nucleus in three fish species from Rajamangala Beach and Libong Island.

Stations	Abnormalities of nucleus	*Zanarchopterus* sp.	*Gerres filamentosus*	*Leiognathus decorus*
Rajamangala Beach	Kidney nuclei	2.50	1.40	2.67
	Notched nuclei or Lobed nuclei	1.50	3.52	1.50
Libong Island	Kidney nuclei	2.00	3.00	3.00
	Notched nuclei or Lobed nuclei	2.00	2.00	1.67

**Table 4:** Observed percentages of three major types of leucocytes in three fish species from Rajamangala Beach and Libong Island.

Station	Type of leucocyte	*Zanarchopterus* sp.	*Gerres filamentosus*	Leiognathus decorus
Rajamangala Beach	Neutrophil	2.50	2.67	3.00
	Lymphocyte	3.00	4.00	6.00
	Monocyte	–	–	–
Libong Island	Neutrophil	2.60	3.33	3.00
	Lymphocyte	4.60	4.00	5.00
	Monocyte	3.00	2.50	1.00
